# The Trochlear Paradox in Native Knees and Its Potential Impact on Total Knee Arthroplasty: An MRI-Based Correlation Study Investigating the Effect of Varying Posterior Femoral Condyle Angles on the Patellofemoral Joint

**DOI:** 10.3390/jcm13030790

**Published:** 2024-01-30

**Authors:** Timon Röttinger, Leonard Lisitano, Johanna Abelmann-Brockmann, Kim Rau, Nora Koenemann, Annabel Fenwick, Edgar Mayr, Heinz Röttinger

**Affiliations:** 1 Universitätsklinikum Augsburg, Klinik für Unfallchirurgie, Orthopädie, Plastische und Handchirurgie, Stenglinstraße 2, 86156 Augsburg, Germany; leonard.lisitano@uk-augsburg.de (L.L.); johanna.abelmann-brockmann@uk-augsburg.de (J.A.-B.); kim.rau@uk-augsburg.de (K.R.); nora.koenemann@uk-augsburg.de (N.K.); annabel.fenwick@uk-augsburg.de (A.F.); edgar.mayr@uk-augsburg.de (E.M.); 2 München Klinik Neuperlach, Oskar-Maria-Graf-Ring 51, 81737 München, Germany; heinz.roettinger@hotmail.com; 3 Faculty of Medicine, Medical University of Pleven, 5800 Pleven, Bulgaria

**Keywords:** femoropatellar joint, posterior condylar angle, lateral trochlear inclination, total knee arthroplasty, lateral femoral condyle, bisect offset, patella tilt

## Abstract

Background: Since the beginning of total knee arthroplasty, implant alignment has been a central point of discussion. As diverse as the discussed alignment theories are, as uniform is the implant design, which is supposed to be based on the “average knee.” Steady upgrades in prosthesis design and modern alignment theories have achieved improvements. However, knee arthroplasty continues to be burdened by a significant percentage of dissatisfied patients. In current knee arthroplasty, femoral implant alignment is referenced by the dorsal and distal condylar axes. The patellofemoral joint variance is not sufficiently considered. Predominantly dorsal and distal referencing at the femoral condyle determines the postoperative shape of the anterior knee joint. The present study investigated a possible relationship between dorsal and distal joint parameters and the patellofemoral joint. Methods: In this explorative retrospective monocentric study, MRI cross-sectional images of 100 native knee joints were evaluated. By determining parametric correlations according to Pearson, the study investigates whether the independent variables “posterior femoral condyle angle” and “lateral distal femoral angle” are related to “lateral trochlear inclination”, “patella tilt”, and “bisect offset”. Results: The posterior condylar angle significantly correlates with lateral trochlear inclination, patella tilt, and bisect offset. There is a positive correlation with patella tilt and bisect offset but a negative correlation with lateral trochlear inclination. The lateral distal femoral angle did not correlate with the studied parameters. Conclusion: The lateral trochlear inclination decreases with an increased posterior femoral condylar angle. The posterior referencing of the femoral component in total knee arthroplasty simultaneously establishes the shape of the anterior knee joint. Our results indicate that increasing posterior condyle angles significantly correlate with flattened lateral trochlear inclinations in native knees and suggest a systematic biomechanical conflict in total knee arthroplasty.

## 1. Introduction

The knee joint, with its femorotibial and femoropatellar parts, presents an enormous variety of shapes. Apart from extremely dysplastic variants, this individualized knee joint design reliably results in proper joint function. The individual knee joint functions as a complex multivariable system. However, how the individual shape variables relate still needs to be determined.

Various developments in knee arthroplasty attempt to approximate the shape of the natural knee joint. Apart from custom-made implants, which are not yet routinely used, the individual product lines of the various manufacturers are based on an implant-specific standard shape, which closely approximates the average appearance of knee joints. With this practice, good to excellent results are predominantly achieved in total knee arthroplasty (TKA), contributing to the fact that implanting an artificial knee joint is one of the most frequent and successful operations in orthopedics. On the other hand, TKA has been burdened with a constant 20–30% proportion of dissatisfied patients since its beginnings [1,2,3,4]. The alignment of the arthroplasty is considered to significantly impact the function and longevity of the knee joint [5,6]. Initially, discussions focused on anatomical and mechanical alignment [7]. Later, gap-balancing alignment was added [8]. Kinematic alignment has recently gained increasing interest [9]. Despite all efforts, the postoperative unsatisfactory results could not be eliminated.

The standard alignment techniques are based primarily on the coronal plane of view [10]. The reference planes for cutting the knee joint to fit the endoprosthesis are the leg axis, the joint line, the distal condylar line, and the posterior condylar line. The alignment techniques differ by an explicit acceptance or defined deviation of the bone cutting with these reference planes.

Numerous studies indicate that the anterior articular component plays a significant, perhaps even decisive, role in the postoperative outcome after TKA [11]. Matching the shape of the anterior knee joint in its preoperative configuration with the postoperative outcome is not represented in any of the known different alignment techniques. To date, it has yet to be adequately investigated whether and, if so, how the distal and dorsal condylar planes correlate with anterior joint variables.

The present study investigated whether there is a correlation between the lateral distal femoral angle (LDFA) and posterior femoral condylar angle (PCA) on the one hand and the anterior knee joint variables patella tilt (PTilt), lateral trochlear inclination (LTI), and bisect offset (BO) on the other in native knees. The results of this study will then be discussed with their relevance to knee arthroplasty.

## 2. Methods

### 2.1. Aim of the Study

The study aimed to verify whether a correlation between the PCA and the LDFA exists with specific variables of the femoropatellar joint. The anterior knee joint variables studied were LTI, PTilt, and BO.

### 2.2. Patient Collective

This study retrospectively analyzed unilateral MRI cross-sectional images of native knee joints from 100 consecutive patients attending orthopedic consultations. The patients attended a normal, wide-ranging orthopedic clinic with various questions relating to the knee joint. The data were already stored in the hospital’s radiology department’s PACS system (Impax, Agfa Healthcare, Agfa HealthCare GmbH, Konrad-Zuse-Platz 1-3, 53227 Bonn, Germany). To clarify different diagnostic questions, the indication for an MRI examination of the knee joint was given during the presentation in the orthopedic consultation based on medical needs. All knee joints that had undergone joint reconstructive surgery before MRI cross-sectional imaging were excluded from participation in the study. Furthermore, the study accepted only knee images in which MRI was available in a flexion of 10° to 20° according to our standard protocol. The study population was set at 100 patients before enrolment. With this study population, it seemed reasonable to determine significant correlations.

Data were collected using Microsoft Excel from Office 365 (Microsoft, Redmond, WA, USA). SPSS version 28 (IBM, Armonk, NY, USA) was used for subsequent statistical analysis.

### 2.3. Radiological Measurements

**Figure 1:**
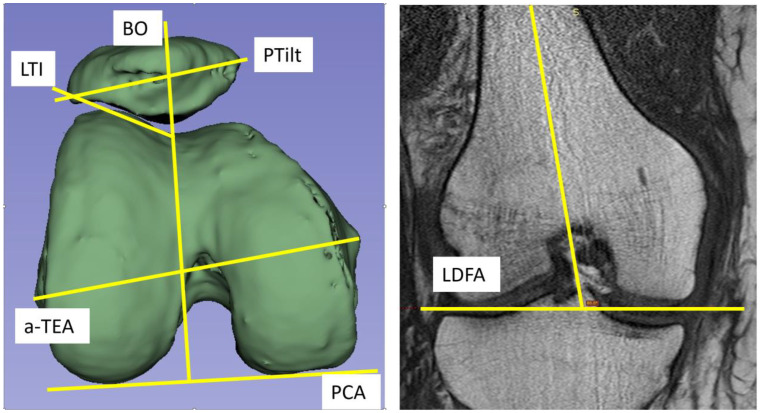
**left:** Typical survey of the measured values. The PCA was measured with reference to the a-TEA. LTI and PTilt angles are referenced to the PCA. The BO is drawn perpendicular to the PCA through the sulcus, dividing the patella into two parts. The index formula BO index = A/(A + B); A = lat. facet, B = med. facet. **right:** Anatomical LDFA (=Lateral Distal Femoral Angle).

LDFA was determined in the coronary plane using the angle of the distal condylar line and the femoral shaft axis (so-called anatomic LDFA). PCA was measured in the axial image with reference to the anatomical transepicondylar line (a-TEA). The measurements of PTilt, LTI, and BO were referenced to the posterior condylar axis (Figure 1) [5,12]. All data collection associated with the imaging was carried out by one of the authors.

### 2.4. Statistical Analysis

The statistical analysis aimed to verify a correlation between the predictors (PCA, LDFA) and the dependent variables LTI, BO, and PTilt. Correlation coefficients were parametrically determined according to Pearson. Beforehand, required conditions were checked. First, boxplots of all variables were created in which the length of the lower and upper whiskers was at most 1.5 times the interquartile range. Outliers outside these whiskers were removed. The normal distribution of the residuals was checked using the Shapiro–Wilk test. Normal distribution was given for all variables. In all tests performed in this study, alpha was set to *p* ≤ 0.05.

### 2.5. Language

Fluently English-speaking authors, some native speakers, wrote this text. This process was also supported by Deepl Translator (licensed version 24.1.1.11641, Deepl SE, Köln, Germany) and Grammarly Premium licensed version, Grammarly Inc., San Francisco, CA, USA).

## 3. Results

Due to the explorative approach, it was impossible to influence the patient collective to be studied. At the beginning of the study, only the total number of involved patients was set at 100 consecutively selected patients. The average age of the 46 female and 54 male patients was 48 years (19–74 years). The results of the study are shown in Table 1.

**Figure 2:**
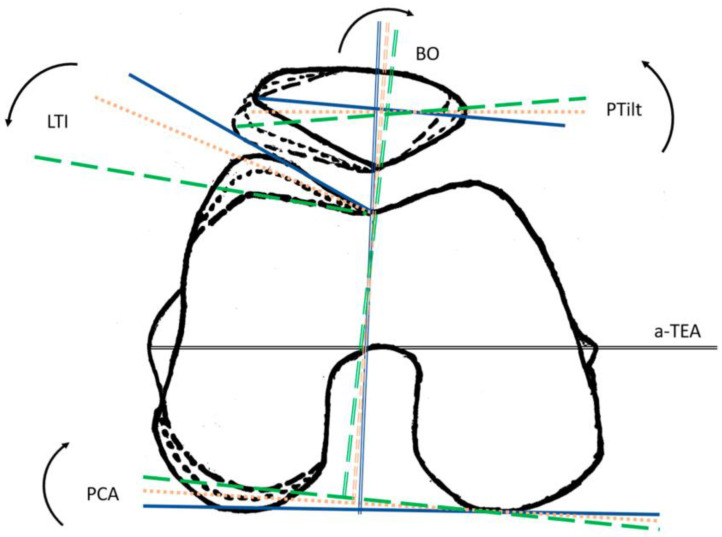
Illustration of PCA (predicter) and LTI, PTilt, and BO (dependent variables) relationships. LTI is negatively correlated with PCA. The change in PTilt and BO is mainly caused by the different trochlea shapes. Increasing PCA leads to a flattened lateral anterior condyle characterized by the LTI and does not rotate in the same direction.

When evaluating the different parameters, a significant positive relationship exists between PCA, PTilt, and BO (Pearson correlation 0.428, respectively, 0.330). A significant negative correlation was noted between PCA and LTI (Pearson correlation −0.319). To clarify, this implies: An increased PCA is more likely to be associated with a decreased LTI. In contrast, as PCA increases, there is also an increase in PTilt and BO. This entire relationship is demonstrated in the schematic illustration in Figure 2. Regarding Figure 2, it should be particularly noted that the same patella shape is involved in each case. Due to the different settings of the patella in the functional system with the varying trochlea shape, there is a change in PTilt and BO without any change in the patella shape.

## 4. Discussion

The present study aimed to verify a correlation between the femorotibial (PCA, LDFA) variables and femoropatellar (LTI, BO, PTilt) joints. The LDFA shows no significant correlations with the variables studied. However, the study revealed significant correlations of the PCA with the LTI, BO, and PTilt, with a negative correlation demonstrated between the PCA and the LTI. The study results could have implications on knee arthroplasty, specifically in terms of transferring these relationships into considerations regarding alignment strategies and implant designs in TKA.

Wiberg’s (in the original paper: Gunnar Wibeeg) publication, which is now mostly associated with patella classification, provides an impressive description of different patella shapes and their impact on the formation of the trochlea. “The articular surface of the patella is in the main an exact cast of the femoral articular surface” [13]. Different patellar shapes will find their counterpart (and vice versa) in the corresponding trochlea.

The mechanical loads in the femoropatellar joint necessitate consistent morphological matching of the direct joint partners throughout the entire range of motion. This condition makes this joint’s wide variety of shapes even more astonishing. Modern cross-sectional imaging facilitates the study of the patella and trochlea’s mutual shape correspondence [14,15,16,17]. The interaction of the BO, LTI, and PTilt has been frequently described. Extreme formations of this coordinated system affect patellar tracking stability [18].

**Figure 3:**
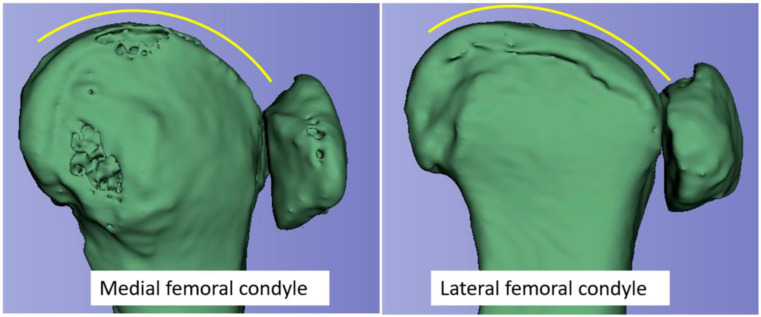
3D animation of the medial and lateral femoral condyles of a knee joint from a CT scan. Note the different shapes of the condyles in the anterior and the transition area to the distal region.

Typically, as seen in the skyline Laurin view, the patella aligns perfectly with the trochlea. A longitudinal examination of the femoropatellar joint, conducted by Krakow and Hungerford, confirmed that the patella contacts an almost flat surface with the corresponding lateral femoral condyle in the sagittal plane [19]. This means that, after flexion of about 30°, the lateral patellar facet slides out of the trochlea to a flattened plane of the lateral femoral condyle (transition area of trochlea to distal condyle) [20]. The different shape of the medial and lateral femoral condyle in the sagittal plane is shown in Figure 3.

With increasing flexion, the patella loses contact with the trochlea and increasingly covers the lateral femoral condyle with the lateral patella facet. In contrast, the patella loses contact with the medial femoral condyle. In maximum flexion, the medial articular surface of the patella is in contact with the medial femoral condyle only through the odd facet. Ficat and Hungerford have described this patella movement in the coronal plane during flexion as a “gentle curve with a concavity facing laterally” [21].

Historically, the study of the patellofemoral joint has been characterized mainly by the clarification and treatment of patellar instability [22]. Over time, this led to an isolated view of the patellofemoral and femorotibial joints. While sports medicine specialists focused heavily on the femoropatellar joint, arthroplasty specialists directed more attention to the femorotibial joint.

Since the beginning, knee arthroplasty has been afflicted with a persistent problem attributed to the patella [5,23]. Approximately 15–25 percent of operated patients complained of troublesome anterior knee pain. All previous attempts to clarify this problem or to find a solution were unsuccessful. Very early on, the rotational position of the femoral component was suspected to be an essential component of this problem [24].

To understand the changes in the patellofemoral joint during arthroplasty using various alignment methods, one should first visualize the biomechanical situation of the healthy patellofemoral joint.

The extension of the knee joint is actively controlled by the quadriceps muscles. The axis of gravity shifts forward with increasing extension and approaches the patella. Both the contact area and the contact pressure are minimal in this case [21]. During flexion, the line of gravity moves away from the patella towards the center of the knee, even exceeding the center of the knee. The patellar contact pressure increases. By increasing the anterior offset in the femoropatellar joint, e.g., in the case of TKA, the line of gravity remains further away from the patella in full extension. The patella is also permanently in full extension and initial flexion in increased contact pressure in the trochlea. TKA implantation in increased internal rotation also leads to an increase in the LTI due to an increase in the contact pressure of the patella. The patella would run in a more medially curved concave line (“curve with a concavity facing lateral”) during initial flexion. The forced medial patella tracking leads to increased lateral contact pressure. Furthermore, a reduction in PTilt and BO is expected [21,25].

In the native knee joint, the patella has little to no contact with the trochlea in full extension, depending on the length of the patellar tendon. During initial flexion, the patella moves into the trochlea from a slightly lateral position. The contact area between the patella and trochlea progressively increases until approximately 30° of flexion [26]. Reversal of the final rotation (screw home mechanism) occurs via a rollback of the lateral femoral condyle on the convex tibial plateau at initial flexion up to approximately 30°. This results in a considerable reduction in the Q angle and lateral vector forces on the patella [21,25,27,28].

Currently, knee arthroplasty refers to the PCA regarding the rotational position of the femoral component. This reference in knee arthroplasty considers that the PCA directly influences the femorotibial joint but is also related to the shape characteristics of the anterior knee joint, specifically patella tilts, BO, and in a negative correlation to the LTI. A possible relationship between the PCA, posterior condylar length, and trochlear dysplasia (TD) has been described recently by Roger et al. [29].

As shown by Woiczinski et al., internal rotation of the femoral component increases retropatellar stress [30]. Our results could be an explanation for the biomechanical conflict described by Woiczinski. A nonphysiological exaggeration of the lateral femoral condyle can still be expected in the transition area from the trochlea to the distal lateral femur, where typically the surface is flattened (see Figure 3).

Numerous studies indicate that the rotational position of the femoral component has a significant influence on patella tracking. The increase in the internal rotation of the femoral component in TKA results in an increased patellar tracking pressure, which must ultimately be attributed to the increased LTI [6,11,30,31,32].

Recent studies also attribute significant function-controlling and proprioceptive properties to the patellofemoral joint component and its adjacent soft-tissue structures [16]. Gross alterations of these control mechanics and patellar tracking could explain the frequently observed feeling of tightness, anterior knee pain, and dystrophic disorders with excessive tissue formation, including arthrofibrosis.

**Figure 4:**
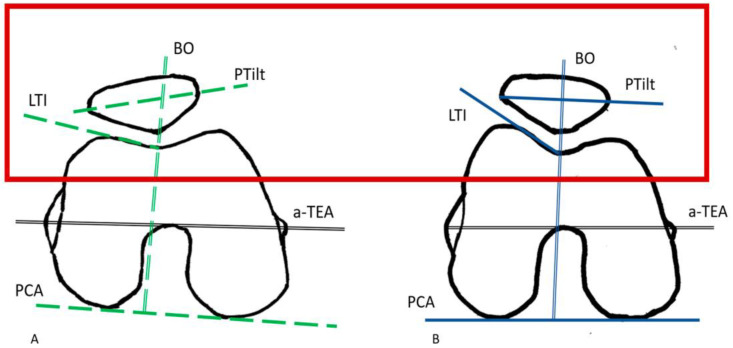
(**A**,**B**): The key points of the illustration in Figure 2 are compared separately. (**A**) shows a sketch of a knee joint with significantly increased PCA and reduced LTI according to the trend results of the present study. Assumptions: In example (**A**), a TKA is inserted under posterior referencing. The contour of the anterior knee joint (delimited by the red frame) changes in the direction of (**B**). The LTI increases. Further results are flattening of the PTilt and medialization of the patella with reduction of the BO.

Knowing the significant correlations between PCA and BO, PTilt, and LTI, one must assume that conflicts will arise through arthroplasty, especially with the extreme morphologies of the anterior knee joint. To illustrate this very specifically, the key points of Figure 2 are compared separately in Figure 4.

Suppose the knee arthroplasty is consistently referenced posteriorly. In that case, there is a potential risk of excessive elevation of the lateral trochlear joint surface and in the subsequent anterior transition region to the distal femur with increasing PCA. With reference to Figure 4A, this would mean that the initial situation for the anterior knee joint (indicated by the red frame in Figure 4) would change in the direction of 4B after TKR. This can be seen on the X-ray by a flattening of the PTilt and a medialization of the patella via a reduction in the BO. As described above, this results in an overall increase in lateral contact pressure on the patella. The extent to which compensation options are available in principle and from a patient-specific, individualized perspective remain open for the time being. Further research and studies are required to clarify this.

## 5. Conclusions

The present study describes the relationships between the PCA and trochlear shape parameters LTI, BO, and PTilt. Especially the variation in the PCA may lead to a mismatch in the anterior knee articulation by TKA. According to our study, increasing PCA appears with a flattened lateral anterior condyle characterized by the LTI. Although plausible, the problem in translating these correlations to arthroplasty has yet to be supported by clinical results. Here, further studies are needed to address the question of the extent to which different PCA or primary trochlear shapes influence the postoperative outcome after the insertion of a TKA.

## Figures and Tables

**Table 1:** The values collected during the study. LTI is negatively correlated to PCA. Std Dev. = standard deviation.

Variable	N	Average	Std-Dev.	Min	Max	LDFA			PCA		
LDFA [°]	94	82.35	1.81	78.3	86.6	Pearson correlation	two-side *p*-value	N	Pearson correlation	two-side *p*-value	N
PCA [°]	100	6.49	2.02	2.4	10.6						
LTI [°]	99	19.58	3.87	10	28	0.129	0.219	93	−0.319	0.001	99
PTilt [°]	94	10.31	4.04	1	18	−0.100	0.350	89	0.428	<0.001	94
BO [%]	94	58.20	6.00	45.6	73.8	0.013	0.902	88	0.330	0.001	94

## Data Availability

The datasets used and/or analysed during the current study are available from the corresponding author on reasonable request.

## List of Abbreviations

TKA	Total knee arthroplasty
TKR	Total knee replacement
LDFA	Lateral distal condylar angle
PCA	Posterior condylar angle
PTilt	Patella tilt
LTI	Lateral trochlear angle
BO	Bisect offset
TEA	Transepicondylar line
a-TEA	Anatomical transepicondylar line
